# Spanning three decades: global research wave and future prospects of broader autism phenotype—a visual researches by CiteSpace and VOS viewer

**DOI:** 10.3389/fpsyg.2024.1453140

**Published:** 2024-12-19

**Authors:** Fu-Qiang Qiao, Si-Ning Li, Tong-Tong Du, Wen-Ming Cheng, Ying-Ying Sun, Xu Qiang, Ya-Jie Dong, Lei Wang

**Affiliations:** ^1^School of Education and Psychology, University of Jinan, Jinan, China; ^2^Department of Wei Zhong Children’s Rehabilitation Center, Jinan, China; ^3^School of Philosophy and Social Development, Shandong University, Jinan, China; ^4^Department of Psychology, Ningbo University, Ningbo, China; ^5^Tianjin Yang Jialou Senior High School, Tianjin, China; ^6^Department of Rehabilitation Medicine, Hunan Provincial People’s Hospital, The First Affiliated Hospital of Hunan Normal University, Changsha, China

**Keywords:** broader autism phenotype, bibliometric analysis, CiteSpace, research trends, web of science, VOS viewer, visual analysis

## Abstract

**Objectives:**

To conduct a comprehensive review of the literature pertaining to the broader autism phenotype, the paper endeavors to delineate the key research directions and topics, document the current research trends, and furnish insightful analyses and novel perspectives to foster future advancements in the field, with the aid of CiteSpace and VOS viewer.

**Methods:**

CiteSpace and VOS viewer are two kinds of software for visualizing citations that is intended to examine academic literature and identify possible sources of knowledge. The Web of Science Core Collection database was used to retrieve articles from 1994 to 2024 that discussed the autism phenotype in general. Following data collection, analyses were conducted using CiteSpace V.6.2.R4 (64-bit) on a number of topics, such as annual publication output, highly cited journals, affiliations with countries and institutions, eminent authors, cited references, and keywords. Knowledge maps, collaborative network analysis, cluster analysis, and strongest citation burst analysis were among the tools used to visualize the data.

**Results:**

A total of 1,075 articles about the broader autism phenotype were found. Roughly speaking, the annual number of publications is rising. With 546 articles on the subject, the United States is the nation with the greatest amount of authority and influence (centrality of 0.32), with England (218 articles) and Canada (115 articles) coming in second and third, respectively. The cited journals and institutions are mainly from the United States. The research consistently emphasizes the academic achievement and engagement of broader autism phenotype when analyzing the most cited references and authors.

**Conclusion:**

This study used CiteSpace to analyze the state of the larger autism phenotype field and then identified research frontiers and hotspots. As new findings are made, global trends in more thorough studies of the autism phenotype suggest that interest in these studies will only grow.

## 1 Introduction

The broader autism phenotype (BAP) is a subclinical set of personality and other features that is thought to index familiarity and/or genetic liability to autism (Lainhart et al., 2002). This view suggests that BAP is milder but qualitatively similar to the diagnostic autism phenotype. A tendency toward rigidity (lack of interest in and/or difficulty adjusting to change) and/or hypersensitivity (excessive distress at remarks or behavior of others that is felt to be critical or insensitive), abnormalities in speech, and qualitatively impaired friendships were considered criteria for the BAP (Lainhart et al., 2002). BAP traits were first observed by Kanner (1943), he implicated parents as a potential cause for autism, claiming that most parents of the children studied did not seem exceptionally warm to their children. Twin and family studies have confirmed that the development of autism is highly heritable (Bolton et al., 2010; Bailey et al., 1995). The twin study of autism by Folstein and Rutter (1977) was the first systematic study to suggest the existence of a broad autism phenotype. Many studies have been done on the impact of the parents’ broader autism phenotype, which is a collection of subclinical traits of autism spectrum disorders, on the child’s autistic disorder phenotype (Wheelwright et al., 2010). As of right now, no single paradigm can adequately explain the nature of BAP and its rising incidence. Several studies have confirmed that the broad phenotype is mainly characterized by cognitive deficits, social deficits, stereotypical behaviors, but also by anxiety, depression, hypersensitivity to criticism, poor adaptability, and over-responsiveness, with social deficits appearing to be more important (Baron-Cohen et al., 2001; Sung et al., 2005; Hurley et al., 2007; Kanne et al., 2012). In conclusion, the above studies confirm the existence of broad autism phenotype and that some relatives of autistic pre-diagnostics have subclinical symptoms that resemble autistic-like manifestations.

Bibliometrics is a quantitative approach to analyzing scholarly publications, which has been applied in many areas of research to assess the patterns of authors, institutions, journals, countries and keywords et al. associated with particular types of publication (Liang et al., 2017). It is also a good choice for identifying research trends and knowledge gaps of a research field over time (Wu et al., 2021). CiteSpace, as one of visualization analysis software, is widely used to explore the research hotspots, research frontiers, knowledge base, main authors and institutions of a research field, as well as to help predict the future direction of a research field (Chen et al., 2022). However, there is a lack of summary and evaluation of the characteristics of the literature, research directions, depth of research, and research hotspots in BAP research. Therefore, there is a need to determine the current state of BAP to inform future researches. In this study, it was aimed to analyze the global research trends and prospects on the BAP last 13 years, CiteSpace was applied to make a bibliometric analysis of related articles collected from Web of Science Core Collections database from 1994 to 2024. Patterns of research publications in this field were mapped to author, institutions, journals, countries, keywords, references, research themes, research hotspots and emerging research areas regarding BAP.

## 2 Methods

### 2.1 Data source

As the independent global citation database for the most reputable publishers in the world, Web of Science (WoS) is the premier research platform for information in the natural sciences, social sciences, arts, and humanities (Luo et al., 2021; Zhou et al., 2021). Additionally, papers that are part of the Web of Science Core Collection (WoSCC) are regarded as being crucial to the research process (Wu et al., 2021). For this study, we chose WoSCC database as our primary data source.

### 2.2 Search strategy

**Figure 1:**
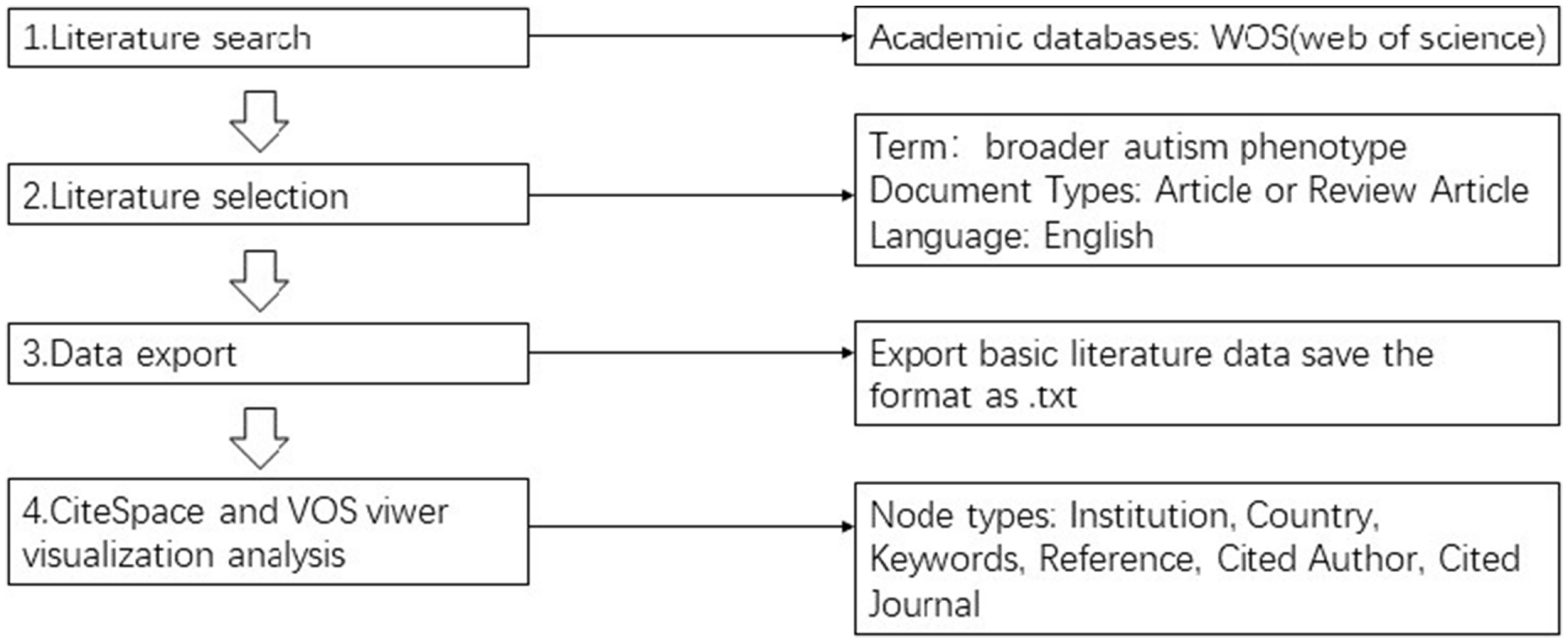
The flowchart of data selection.

We collected data on WoSCC on October 31, 2024.The four components of the data retrieval strategy were as follows: The topics are “broader autism phenotype,” “article” or “review article,” and “language” is “English.” Complete records were downloaded in plain text format for additional analysis, along with the cited references that corresponded with them. In Figure 1, the flowchart is displayed.

#### 2.2.1 Inclusion criteria

Inclusion criteria were: (i) articles on BAP, limited to English as a language; (ii) articles taken from the WoSCC database.

#### 2.2.2 Exclusion criteria

The following were the exclusion criteria: (i) hand-and telephone-collected articles, or newsletters, notices, announcements, calls for papers, and conference papers; (ii) articles not written in English; (iii) conference proceedings and abstracts; (iv) duplicate publications or the same study; (v) unpublished articles and unrelated articles.

### 2.3 Analysis tool

CiteSpace, a bibliometric analysis software developed by Chen (2004), is a citation visual analysis tool that enables the exploration of knowledge potential in scientific literature and gradually evolves under the background of scientometrics and data visualization (Gao et al., 2022). This study utilized CiteSpace V.6.2.R4 (64-bit) to analyze relevant research on BAP, The objective was to provide evidence-based support for educators and researchers, gain insights into the current state and trends in the field, and generate new ideas for future development.

VOSviewer is a free and open-source software for visualizing and analyzing scientific literature, keywords and author relationships, developed by Leiden University in the Netherlands. It is a knowledge graph visualization tool mainly used to build and view bibliometric knowledge graphs. The software is based on the principle of co-citation and co-citation of literature, has strong visualization capabilities, is suitable for large-scale sample data, and supports a variety of view browsing methods, such as label view, density view, clustering view, and dispersion view.

R language was originally developed by two people from the University of Auckland, New Zealand, Ross Ihaka and Robert Gentleman, R language has a wide range of applications, this paper focuses on the application of R language for data analysis and visualization, statistical analysis, data mining, machine learning and other work, and through a variety of charts and visualization means to show the results of data analysis.

### 2.4 Data analysis

CiteSpace software was used in this study to find the citation bursts in a number of different dimensions, such as hotspots, keywords, research publication year, author, research institution, journal, and country. With the help of CiteSpace, one may create a visual knowledge network made up of nodes and connections. The nodes stand for various aspects including authors, countries, institutions, and cited references, while the linkages connecting the nodes show collaboration or co-citation relationships. Nodes’ sizes indicate how frequently or how much they occur, and different colors stand for different years—lighter hues denoting more recent years, and darker colors representing earlier years. Purple circles also indicate centrality. High centrality nodes are frequently seen as pivotal or turning points in the field (Liu et al., 2019).

## 3 Results

### 3.1 Annual publication analysis

**Figure 2:**
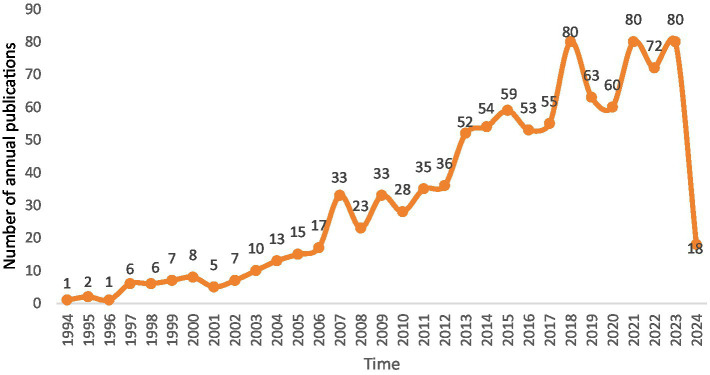
The number of publications from 1994 to March April 2024.

After applying the search strategy, 1,075 publications meeting the inclusion criteria were found; Figure 2 displays the annual number of articles published. It’s evident that 2018, 2019 and 2023 are the years with the most publications. The number of publications has been trending in a fairly stable direction in recent years. The fact that the majority of the publications in this field were published within the previous ten or so years further indicates how new this field is.

### 3.2 Countries analysis

**Figure 3:**
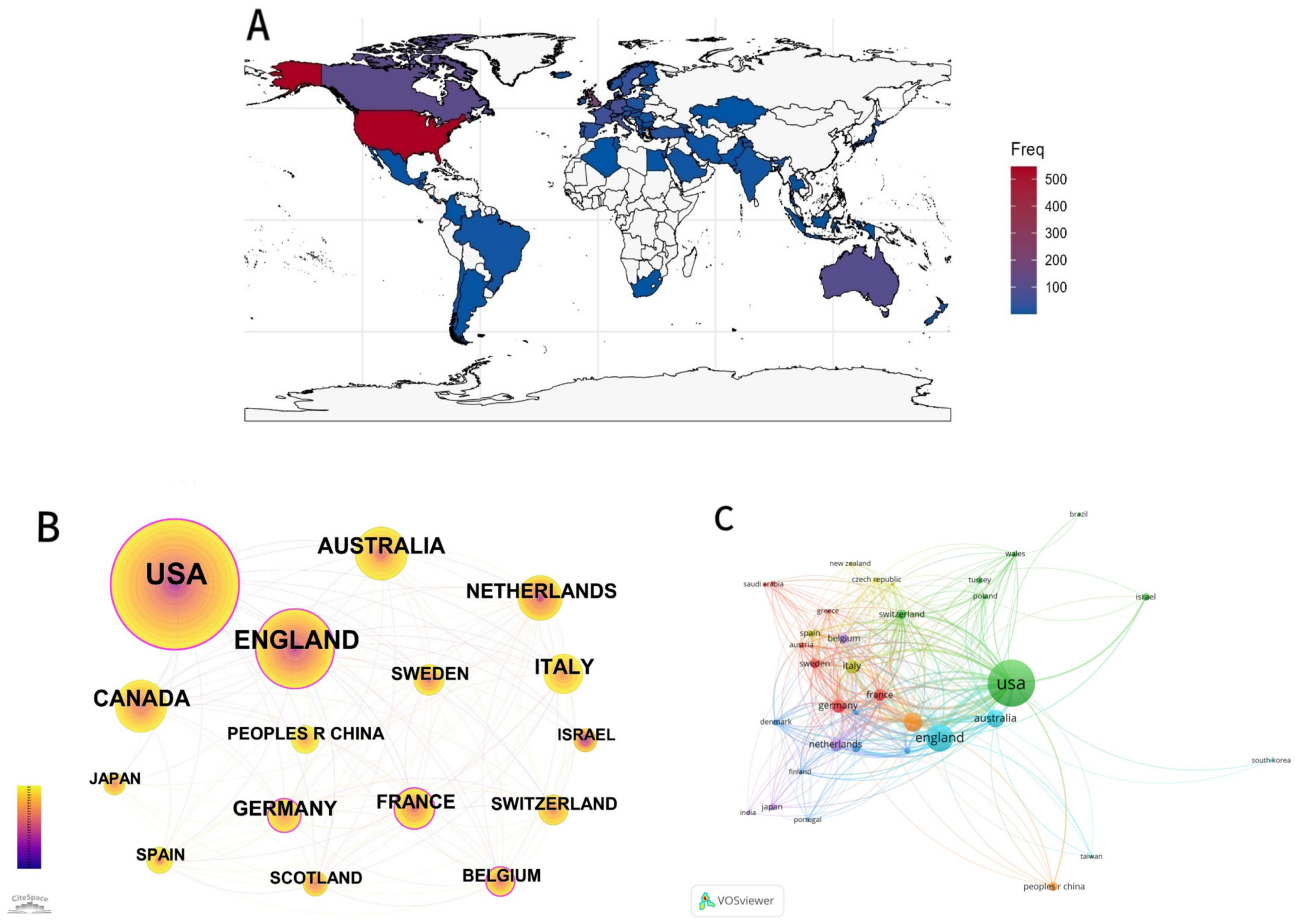
Contributions from various nations/regions to the broader autism phenotype. **(A)** Regional map of the broader autism phenotype studies’ global spread. Different nations are represented by various hues depending on the number of articles published. More articles have been published when the color is redder; fewer articles have been published when the color is bluer. Because the map program cannot search for specific regions, we have provided the country in which the area is located directly. **(B)** A network diagram based on the study of country/regional collaboration and time-course-visualized using Cite Space. **(C)** A network diagram based on the study of country/regional collaboration and time-course-visualized using VOSviewer.

As shown in Figure 3A, these publications come from more than 70 countries/regions. The top ten countries/regions produced 99.16% (1,066/1075) of the publications, with USA (50.79%, 546/1075), the England (20.27%, 218/1075), and Canada (10.69%, 115/1075) leading the way. We used 1-year slices to analyze all BAP articles published between 1994 and 2024 in order to look into the relationships between articles published in various countries. A merged network with 70 nodes and 541 links, representing a country-specific distribution map, was produced by this analysis and is shown in Figure 3B. The differences in the number of publications of each country can also be seen relatively directly from the size of the circles in Figure 3C. Countries are represented by the nodes, and their relationships are shown by the lines. Larger nodes indicate higher publication productivity. The size of the nodes reflects the number of publications. Table 1 lists the twenty most productive nations in the world in terms of BAP.

**Table 1:** The top twenty countries and centrality in the research field of BAP from 1994 to 2024.

Ranking	Countries	Frequency	Centrality
1	USA	546	0.32
2	ENGLAND	218	0.14
3	CANADA	115	0.07
4	AUSTRALIA	103	0.03
5	ITALY	73	0.04
6	GERMANY	65	0.14
7	NETHERLANDS	61	0.05
8	FRANCE	54	0.12
9	SWEDEN	38	0.03

As we can see, the United States is leading all other countries by a significant margin and has the highest publications productivity (546). It is also the only nation with over 200 articles. England, Canada, Australia, and Italy came next.

### 3.3 Cited journals analysis

**Figure 4:**
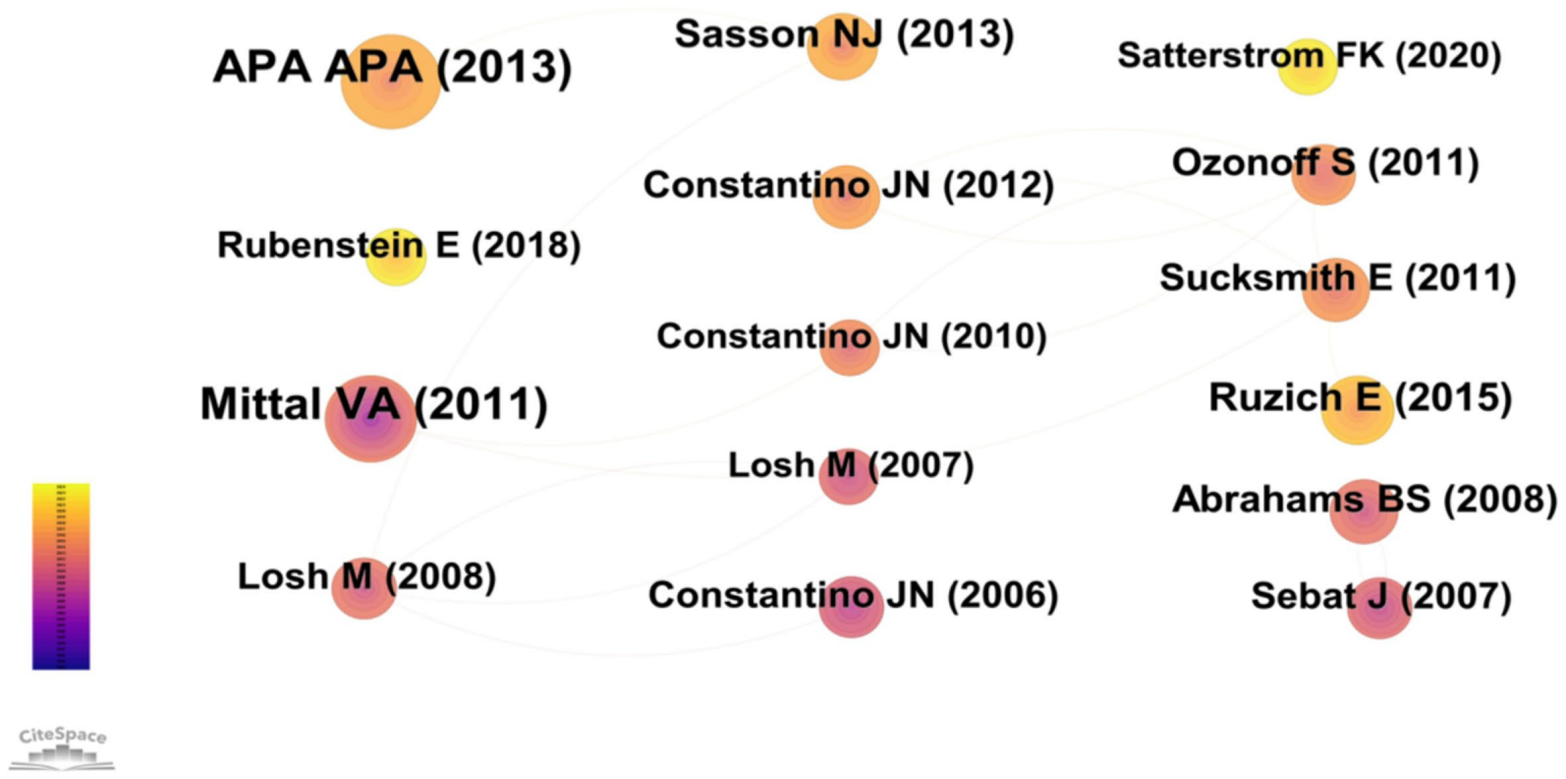
A dual-map overlay of the journals of BAP research.

Through an examination of the journals cited in the field of BAP, we can acquire valuable insights into the primary sources of knowledge dissemination and effectively identify pertinent data. We used 1-year slices to analyze all BAP articles published between 2011 and 2024, classifying the cited journals as nodes. This resulted in a distribution map of cited journals with a merged network that had 1,260 nodes and 5,392 links, as seen in Figure 4. Journals are represented by nodes, and the relationships between journals are shown by lines. Higher frequency nodes are typically regarded as significant nodes that have a bigger influence on how a scientific field develops. A tighter relationship is indicated by a thicker line connecting two nodes.

### 3.4 Institutions analysis

In order to examine cooperative relationships between institutions and pinpoint significant institutions in the field of BAP, we created a network map using Cite.

#### 3.4.1 Space

**Figure 5:**
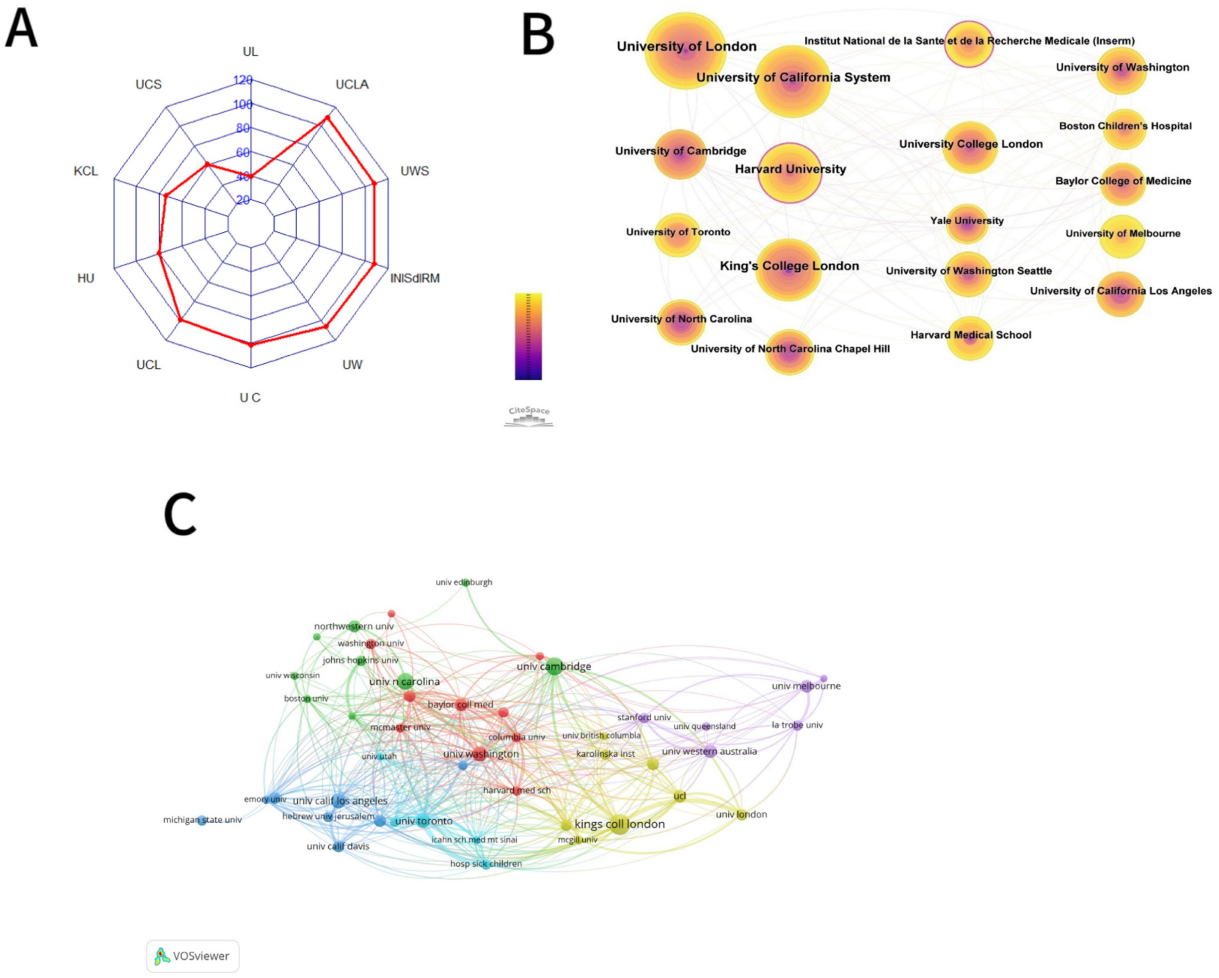
Visual analysis of institutes and funds. **(A)** Frequency radar map of the top ten funds. **(B)** Co-occurrence map of institutions of BAP from 2011 to 2024. **(C)** Visualization of the frequency of publications at each university.

Upon selecting “institution” as the node type, we were able to generate an institutional distribution map (see Figure 5) that showed a combined network with 427 nodes and 2,539 links. Institutions are represented by nodes, and the relationships between them are shown by lines. Nodes that have published more papers are typically regarded as significant organizations that have a bigger influence on the advancement of a scientific field. A closer relationship is indicated by a thicker line connecting two nodes. Table 2 displays the top fifteen most productive institutions in this field of study. With 101 publications, the University of London is the most published institution overall. University of California System is ranked second with 79 publications, while King’s College London is ranked third with 66 publications. The United States holds a prominent position in the field as evidenced by the majority of the top ten universities being situated within its borders. Furthermore, it is evident that there is a strong connection between the major institutions.

**Table 2:** The top ten institutions and centrality in the research field of BAP from 2011 to 2024.

Ranking	Institutions	Frequency	Centrality	Half-life
1	University of London	101	0.09	17.5
2	University of California System	79	0.1	19.5
3	King’s College London	66	0.05	17.5
4	Harvard University	60	0.12	18.5
5	University College London	41	0.05	13.5
6	University of Cambridge	39	0.06	17.5
7	University of Washington	34	0.04	13.5
8	Institut National de la Sante et de la RechercheMedicale (Inserm)	32	0.11	10.5
9	University of Washington Seattle	32	0.03	13.5
10	University of California Los Angeles	31	0.04	11.5

### 3.5 Authors analysis

We employed a 1-year time slice for our analysis and chose articles that were released between 1994 and 2024. We created a co-authorship network map in Cite.

**Figure 6:**
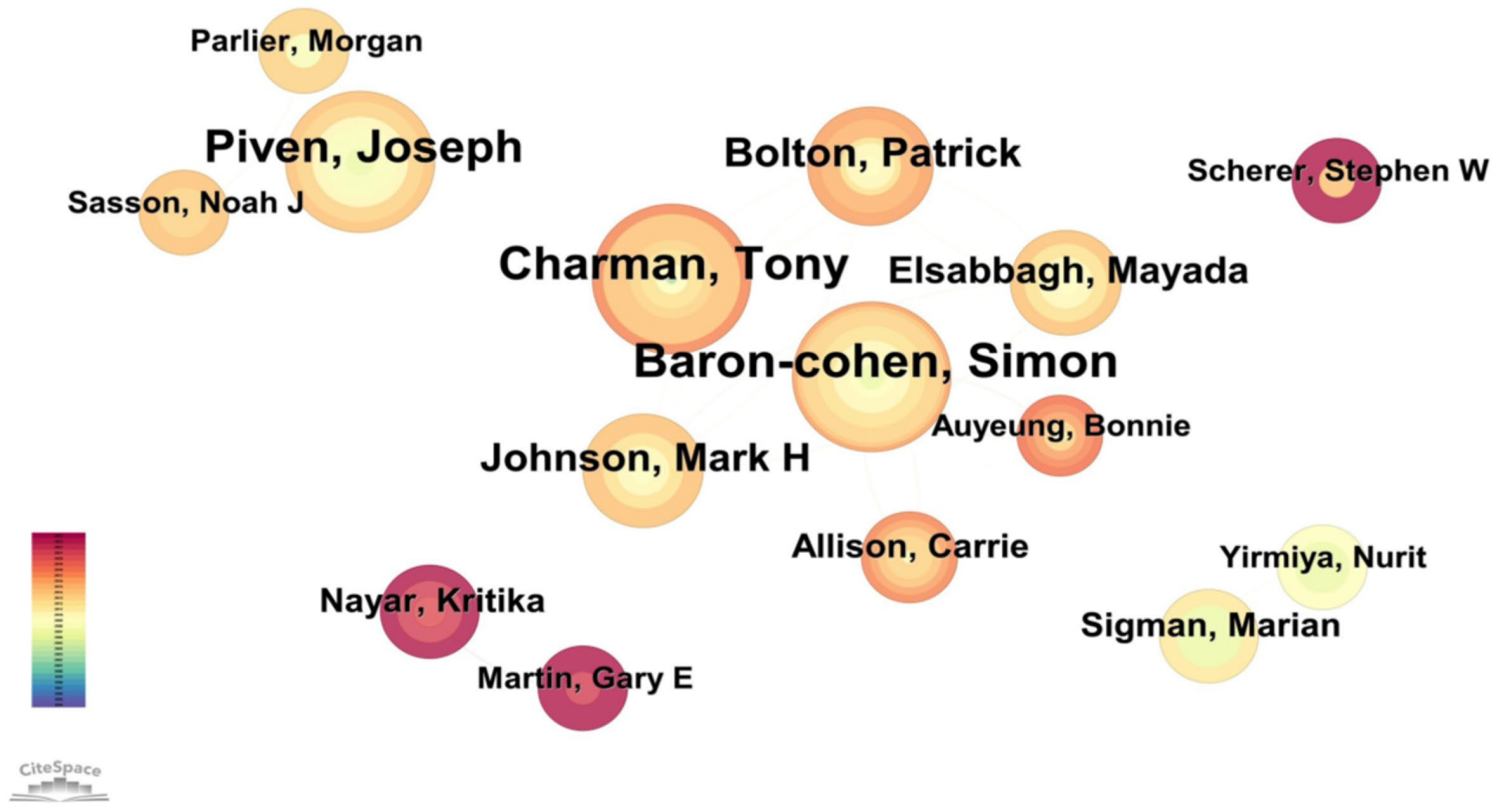
Co-occurrence map of author of BAP from 2011 to 2024.

Space by selecting authors as the node type. As shown in Figure 6, the combined network had 720 nodes and 1,343 links. Authors are represented by nodes, and author relationships are depicted by lines. In the collaboration Figure 6, the author’s output is represented by the size of the node; the larger the node, the more articles the author has published. The degree of collaboration between them is indicated by the thickness of the line that separates them. Baron-cohen and Simon, the active authors from University of Cambridge’s Departments of Experimental Psychology and Psychiatry, ranks first with 18 publications. Charman and Tony from University College London’s psychology department comes in second with16, and Simon Baron-Cohen from the University of Cambridge’s Autism Research Center comes in third with 15 publications. Table 3 displays the top ten authors in this field of study in terms of productivity. The writers are closely related to one another, and many of them have training in psychology.

**Table 3:** The top ten author and centrality in the research field of BAP from 2011 to 2024.

Ranking	Author	Frequency	Centrality	year	Half-Life
1	Losh, Molly	18	0.01	2007	13.5
2	Baron-cohen, Simon	16	0.01	2006	5.5
3	Charman, Tony	15	0.04	2003	9.5
4	Piven, Joseph	14	0.02	2007	2.5
5	Dell’osso, Liliana	10	0	2020	1.5
6	Bolton, Patrick	10	0.02	2009	5.5
7	Johnson, Mark H	9	0	2009	2.5
8	Carpita, Barbara	9	0	2020	2.5
9	Elsabbagh, Mayada	8	0	2009	2.5
10	Rutter, M	7	0	1995	2.5

A more accurate representation of the discipline’s general trend can be seen in tables that examine the core authors’ research directions and number of publications. Price’s (1969) formula is applied here to determine a candidate for core authors. The equation is:

A research technique called co-citation is used to quantify the relationships between articles when one or more papers simultaneously cite two or more articles, a relationship known as co-citation (Zhou et al., 2021). In CiteSpace, we chose references as the node type for our analysis, and we set a 1-year time slice for the articles’ publication years between 1994 and 2024. This produced a co-citation network map, which, as shown in Figure 6, showed a merged network with 642 nodes and 2,631 links. Co-citations are represented by nodes, and the relationships between co-citations are shown by lines. Table 4 displays the ten most cited references in this study’s film. Erratum: Ruzich et al. (2015) most-cited work is Measuring Autistic Traits in the General Population: a systematic Review of the Autism-Spectrum Quotient (AQ) in a Nonclinical Population Sample of 6,900 Typical Adult Males and Females (Ruzich et al., 2015). The Broad Autism Phenotype Questionnaire: Prevalence and Diagnostic Classification was written by Sasson et al. (2013) came next, and followed Recurrence Risk for Autism Spectrum Disorders: A Baby Siblings Research Consortium Study, authored by Ozonoff et al. (1994). It is evident that the majority of the co-citations discuss academic achievement, academic performance, and engagement.

**Table 4:** The top ten cited reference and centrality in the research field of BAP from 1994 to 2024.

Ranking	Cited reference	Year	Frequency	Centrality
1	Diagnostic and statistical manual of mental disorders, fifth edition, and the impact of events scale-revised	2013	67	0.01
2	Diagnostic and statistical manual of mental disorders	1994	64	0.01
3	Measuring autistic traits in the general population: a systematic review of the Autism-Spectrum Quotient (AQ) in a nonclinical population sample of 6,900 typical adult males and females	2015	37	0.02
4	The Broad Autism Phenotype Questionnaire: Prevalence and Diagnostic Classification	2013	35	0.01
5	Advances in autism genetics: on the threshold of a new neurobiology	2008	32	0.02

### 3.6 Keywords analysis

**Figure 7:**
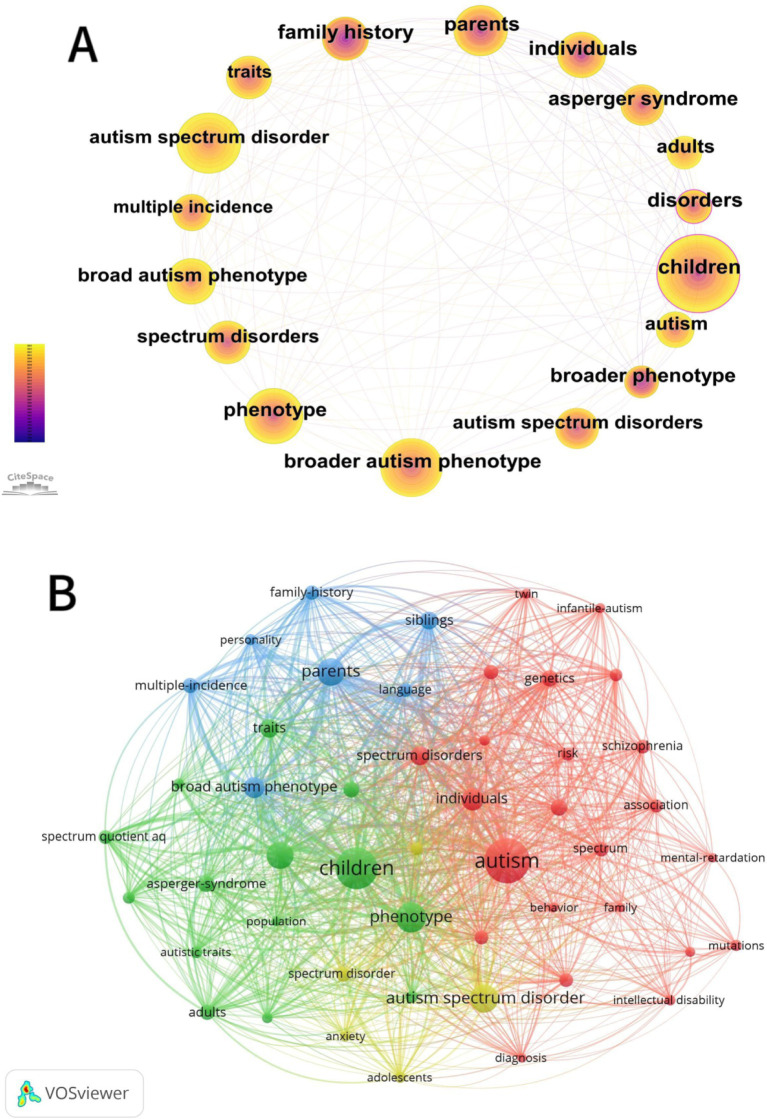

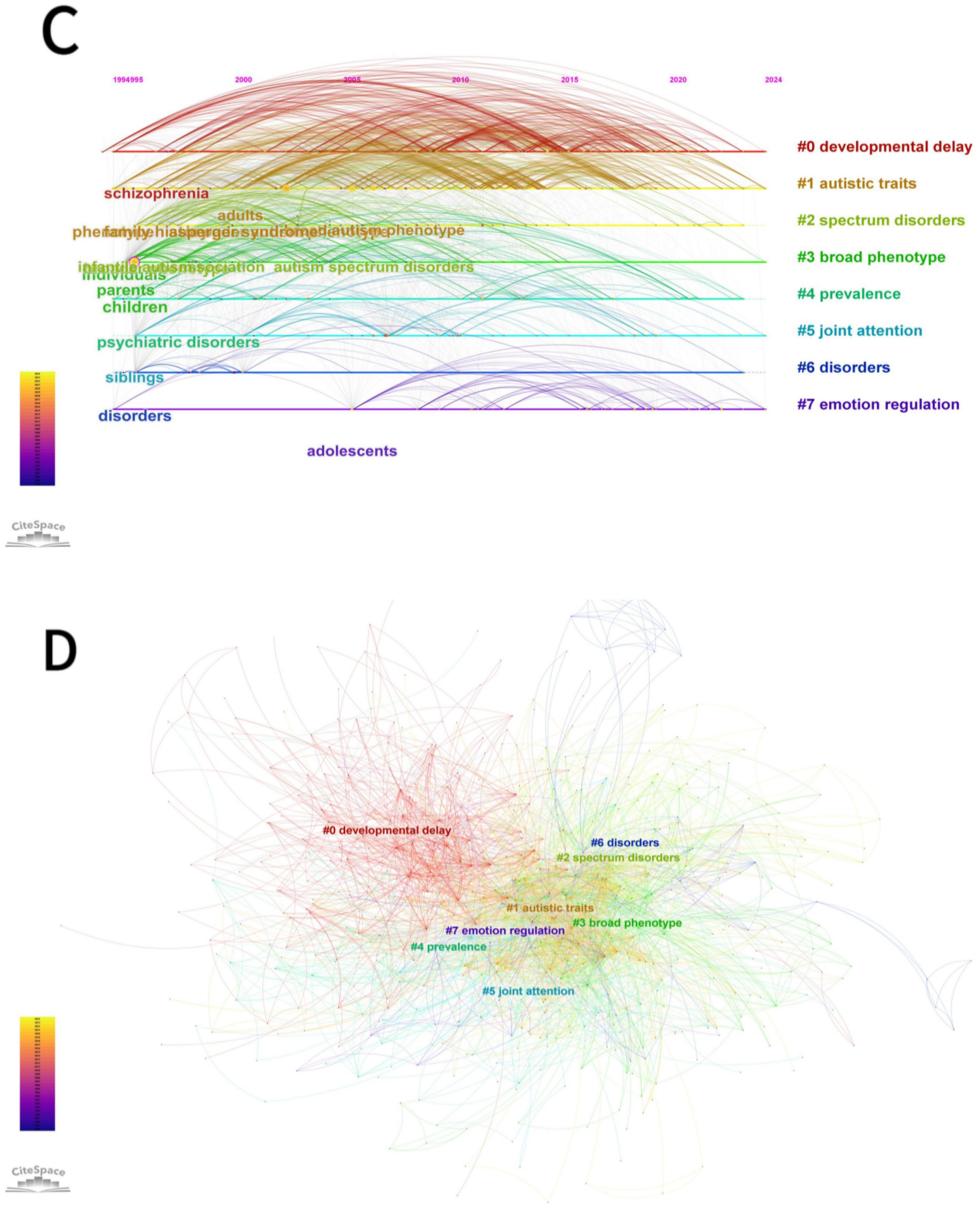
Visual map of keywords of BAP from 2011 to 2024. **(A)** The co-occurrence map of keywords. **(B)** Keywords co-occurrence analysis. Each keyword appeared at least 14 times. **(C)** Co-occurrence map of keywords timeline of BAP from 2011 to 2024. **(D)** The clustering map of keywords.

Keywords are extremely concise and broadly defined, making it possible for readers to rapidly understand the main ideas of the study, This helps with hotspot analysis and development trend prediction. Understanding the core of a study requires an understanding of its keywords. We can find hotspots, examine research directions, and summarize the research topics in a particular field by examining keywords (Ma et al., 2021). A one-year time slice of papers published between 1994 and 2024 was chosen for our analysis, and keywords were selected as the CiteSpace node type. As seen in Figure 7, this produced a term co-occurrence map that showed a combined network with 584 nodes and 4,840 linkages. The study’s most frequently occurring keywords were children (215), autism spectrum disorder (155), broad autism phenotype (140), phenotype (124), broad autism phenotype (98), parents (91), individuals (80), traits (73), and spectrum disorders (69).

**Figure 8:**
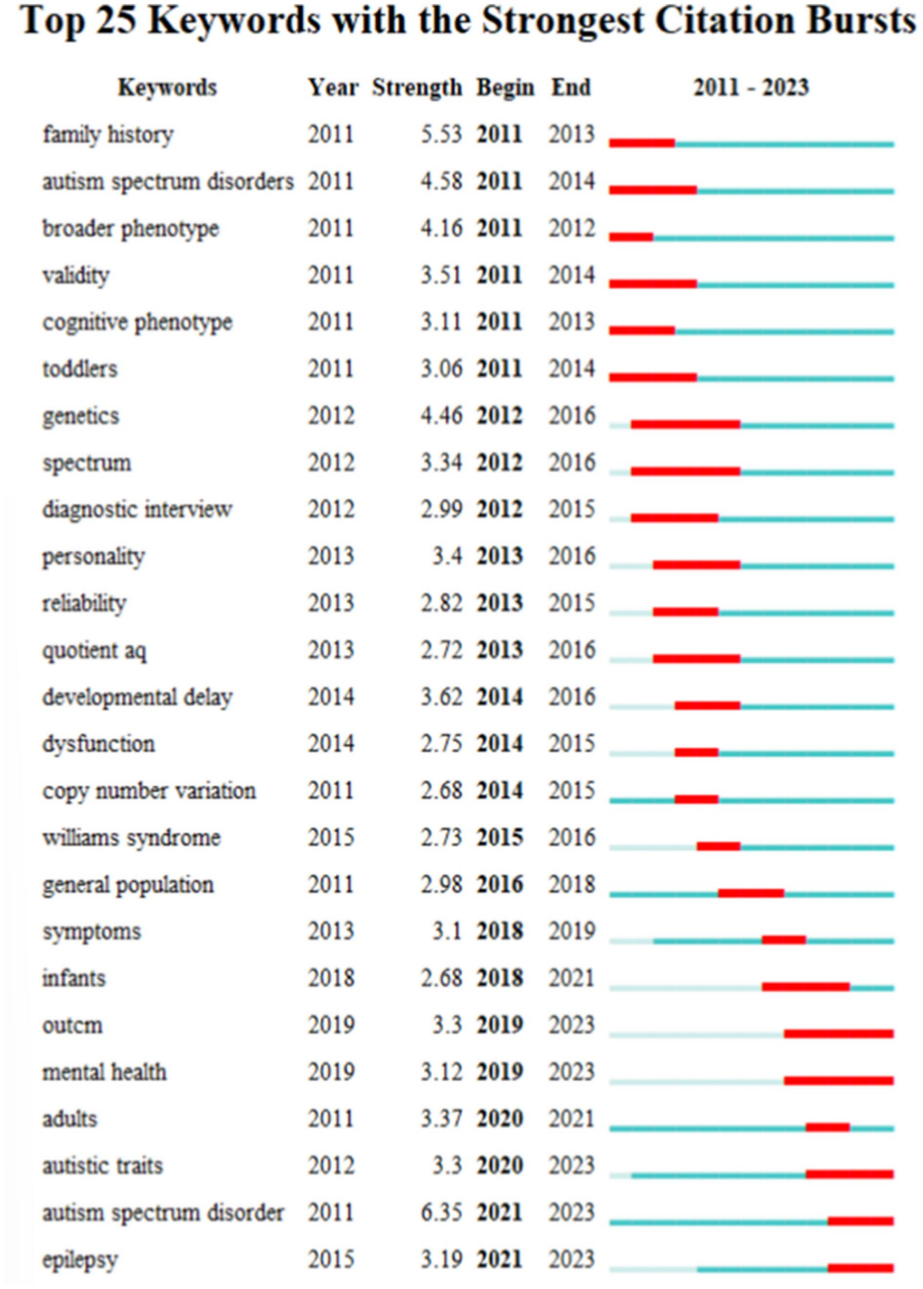
Visualization of top 25 keywords with the strongest citation bursts of BAP from 2011 to 2024.

In order to investigate the evolutionary trajectory and stage characteristics of the research field the timeline is predicated on the interactions and mutation relationships between keywords in a particular field. The co-occurrence map of keywords in the BAP timeline from 1994 to 2024 is shown in Figure 7 which also highlights hotspots and development directions in this field over time. The top 25 keywords with the most powerful citation bursts are displayed in Figure 8. The time intervals are shown by the blue line and the keyword outbreak time is indicated by the red line. The most recent terms to appear in the field of BAP research are epilepsy and autism spectrum disorder (after 2021). Earlier keywords in the field include family history autism spectrum disorders broader phenotype validity cognitive phenotype and toddlers (before 1994). Furthermore there is a lot of strength in genetics spectrum mental health and other factors. Future BAP trends may include the general population symptoms infants and adults

In the formula proposed by Donohue (1973):


$$T=\left[-1+\surd \left(\left(1+8I\right)\right)\right]/2.$$

T is the threshold of high-frequency keywords, and I is the total amount of keywords. High-frequency words can be easily located by computing the threshold (Kee and Tang, 2016). CiteSpace’s metadata indicates that *I* = 215, from which *T* ≈ 20.24 is computed, yielding a list of high-frequency keywords with a frequency more than 20.

## 4 Discussion

### 4.1 Global trends on FGCS

A quantitative method of examining academic literature, bibliometrics has been used in numerous fields of study to evaluate the trends among authors, journals, institutions, nations, keywords, and other factors connected to specific publishing kinds. One visualization analytic tool called CiteSpace is frequently used to investigate research hotspots, research frontiers, knowledge bases, primary authors, and institutions in a field of study, and it is useful for forecasting a topic’s future trajectory. It is also a useful option for determining over time the research trends and knowledge gaps in a field of study (Liang et al., 2017; Chen et al., 2022; Wu et al., 2021). Among the 1,075 literature we searched for, the earliest three articles were autistic spectrum disorder traits in children with attention deficit hyperactivity disorder, a cognitive endophenotype of autism in families with a multiple incidence and the relationship between the broader autism phenotype, child severity, and stress and depression in parents of children with autism spectrum disorders. “Autistic Spectrum Disorder Traits in Children with Attention Deficit Hyperactivity Disorder” researches whether autistic-like communication and social difficulties in children with ADHD are part of the broader ASD phenotype or are specific to ADHD. “A Cognitive Endophenotype of Autism in Families with Multiple Incidence” examines the neurocognitive endophenotype of autism, in families with multiple incidence autism and their findings do not confirm the hypotheses of weak central coherence or deficits in theory of mind as part of the broader endophenotype of autism. “The Relationship between the Broader Autism Phenotype, Child Severity, and Stress and Depression in Parents of Children with Autism Spectrum Disorders” examines the relationship between child symptom severity, parent broader autism phenotype (BAP), and stress and depression in parents of children with ASD. The growth trend of BAP publications globally from 1994 to 2024 can be categorized into 3 phases. The first phase is, a slow upward trend from 1994 to 2006, the second phase is a downward trend from 2006 to 2013, and the third phase is a fluctuating trend from 2013 to today. During these 13 years, the highest number of publications was in 2018, 2021 and 2024 which both with 80 articles published. Nevertheless, BAP research lacks a description and assessment of the features of the literature, research orientations, research depth, and research hotspots.

The total number of articles published in the last 13 years has gone through some ups and downs, but has generally shown an upward trend to reach a total of 1,075. A paper with 100 or more citations is typically regarded as a “classic” or possibly even a seminal paper in the field of study. Generally speaking, the highest number of citations per year for a paper usually occurs between 3 and 10 years after publication (Xiong et al., 2021). The highest number of citations in this field of study is Genetic Heritability and Shared Environmental Factors Among Twin Pairs with Autism with 1,541 citations and Recurrence Risk for Autism Spectrum Disorders: A Baby Siblings Research Consortium Study 1,130 citations. Through analysis of the literature, we find that susceptibility to ASD has moderate genetic heritability and a substantial shared twin environmental component and the sibling recurrence rate of ASD is higher than suggested by previous estimates (Hallmayer et al., 1994; Ozonoff et al., 1994). Analyses of the papers indicate that research in this area has focused on ASD, suggesting that the research on ASD is extremely essential when it comes to BAP.

### 4.2 Research focus and hotspots on BAP

Analysis of the results of high-frequency and strongest burst keywords showed that the research focus and hotspots of BAP has changed over time. Since 1994, people have paid more attention to the study of BAP in families, emphasizing the relationship between symptoms of parents and children. Any child intervention plan must take into account the parent and family factors. Understanding that some family members have BAP traits should be a part of overall family considerations when it comes to ASD, especially in families with multiple affected children. A thorough family history taken before treatment begins can also be useful in identifying whether a multiplex family has a higher likelihood of BAP expression and in clinical settings when recommending the best course of action for a particular child (Gerdts et al., 2013). The presence of BAP in parents of ASD children was found to be substantially linked to the child’s phenotypic class, which included mild language and motor delays, average nonverbal abilities, and more co-occurring conditions like anxiety, depression, and sleep issues (Rubenstein et al., 2019).

Meanwhile, BAP research also focuses on cognitive aspects. More strongly than other neuropsychological characteristics of autism, social-cognitive deficits distinguish parents with the “broad autism phenotype” from parents without it. This suggests that this domain may be especially useful for identifying genetic and brain processes linked to the phenotype (Sasson et al., 2013). And cognitive barriers are also age-related. When people with the BAP grew older, their subjective cognitive impairment increased even though they did not experience any other cognitive decline or accelerated memory loss (Caselli et al., 2018). According to a study, about one in two adults and adolescents with the BAP showed a decreased capacity to recognize and understand social faux pas and there were no gender differences in advanced ToM abilities. A key endophenotype in a subset of people with the BAP may be impaired advanced ToM abilities (Green et al., 2020).

The study of ASD has been a hot topic and the latest trend in the study of BAP since 1994. Meanwhile, research on AT has also become a hot topic in BAP in recent years. In a study, researchers used AQ (Autism Spectrum Quotient) to evaluate autistic traits in participants and found that a significant proportion of fathers and mothers of ASD children belonged to the high-scoring group, indicating that BAP was not sensitively detected among parents of ASD children by paying attention to detail (Bora et al., 2017). Studies on the BAP could help those who live with family members who are autistic by offering more support and direction. For instance, fathers of autistic children who exhibit the BAP could be given tips on how to handle and strengthen their relationships with peers and other family members (Sucksmith et al., 1994).

### 4.3 Study strengths and limitations

Through the creation of knowledge maps and timelines, bibliometric analysis can perform visual analysis on bibliographic data, giving academics a better understanding of the connections and development trends among publications. This study examines the global trends and future prospects in BAP research during the previous 13 years through a projected bibliometric analysis of BAP. Additionally, the search was not restricted to a single academic publication but rather used WoSCC as a database to yield rich data thanks to the precise and thorough literature search criteria. The bibliometric analysis of this study also included popular subject categories, keyword analysis, nation and institutional productivity, annual publishing output, the most cited reference, country, institution, and journal distribution.

Ultimately, a few restrictions need to be taken into account. First, we did not use any other electronic databases like PubMed or Embase; instead, we only used the WoSCC data that was available. Consequently, there could be a delay in the papers gathered from the WoSCC database, which would introduce bias into the study’s citation counts and H-indexes. Secondly, the citation analysis could be biased by nature. Citation rates differ according on specialization and the size of the field of study. There are typically more classical references in more popular scientific subjects. Furthermore, authors are more likely to cite their own works, and papers written in English are more likely to be cited in general. Thirdly, the legitimacy or scientific rigor of publications is not adequately taken into account by bibliometric approaches. Publications with a high citation count may not always be of excellent scientific caliber. Finally, since we were just using BAP as a search keyword, it’s possible that some articles were missed. Also, the scope of our analysis was limited to the last 13 years, leaving out older research on BAP, which may have contributed to its incomplete representation.

In conclusion, despite these drawbacks, we still think that scholars can gain a deeper and more thorough understanding of the development trends and research hotspots of publications pertaining to BAP by utilizing CiteSpace for visual statistical analysis.

## 5 Conclusion

This study gathered a significant amount of reliable data by conducting a bibliometric analysis of the BAP literature published in the previous 12 years. It also summarized previous research, replicated the BAP research process, investigated global research hotspots, and evaluated the boundaries of BAP development in the private sector. The study’s findings demonstrate that, over the previous 20 years, there has been a roughly global increase in the total number of papers pertaining to BAP, suggesting that BAP has attracted a lot of attention. Through the analysis of 1,075 articles, we discovered that this field of research has experienced significant growth after 2017, suggesting that it is still a young field with a wealth of untapped research opportunities. The two most popular research areas are sociology and psychology. More than any other nation, the United States is the primary research force in this area. The remarkable thing about the BAP research field is how many countries, institutions, and authors have joined forces to collaborate extensively. Our analysis of the clustering results and keyword frequency in the scholarly literature reveals that the hotspots and focus of BAP research have evolved over time. Some research hotspots are summarized as follows:

1. Numerous phenotypic-focused research hotspots emerged in 1994, examining the relationship between cognitive phenotype, broader phenotype, and autism spectrum disorders. Researchers have increasingly increased the scope of BAP research to include the general population such as infants and adults as well as paying greater attention to the mental health concerns that BAP experience in recent years.
2. The BAP’s academic issues have been a significant area of study for almost 10 years, and the obstacles and problems the BAP faces in the academic setting have garnered a lot of attention.
3. Research on the specific symptoms of BAP (such developmental delay) and how it relates to autistic traits and autism spectrum disorder has become increasingly popular as the field grows.
4. Research trends in the future may concentrate on social support, cultural identity, and other topics in addition to the previously mentioned factors.

In summary, this study presents a historical, forward-looking perspective and provides a comprehensive and reliable body of research that provides an accurate overview of the global landscape of BAP research. Although these records do not.

The seven citations with the most intense bursts (sorted by year of bursts onset). The blue line represents the time when the citation appeared, and the red line is the time when the bursts happened. Encompass every single publication on BAP, they do provide a significant and representative sample that can offer valuable insights into the challenges and opportunities faced by this population.

Overall, this study sheds information on important topics including familial inheritance and particular BAP symptom presentations. Further thorough and in-depth research on BAP is needed.
